# Optimizing Tactics for Use of the U.S. Antiviral Strategic National Stockpile for Pandemic Influenza

**DOI:** 10.1371/journal.pone.0016094

**Published:** 2011-01-19

**Authors:** Nedialko B. Dimitrov, Sebastian Goll, Nathaniel Hupert, Babak Pourbohloul, Lauren Ancel Meyers

**Affiliations:** 1 ORIE Program, University of Texas at Austin, Austin, Texas, United States of America; 2 Integrative Biology, University of Texas at Austin, Austin, Texas, United States of America; 3 Preparedness Modeling Unit, U.S. Centers for Disease Control and Prevention, Atlanta, Georgia, United States of America; 4 Weill Cornell Medical College, New York, New York, United States of America; 5 Mathematical Modeling, British Columbia Centre for Disease Control, Vancouver, British Columbia, Canada; 6 The Santa Fe Institute, Santa Fe, New Mexico, United States of America; University of Hong Kong, Hong Kong

## Abstract

In 2009, public health agencies across the globe worked to mitigate the impact of the swine-origin influenza A (pH1N1) virus. These efforts included intensified surveillance, social distancing, hygiene measures, and the targeted use of antiviral medications to prevent infection (prophylaxis). In addition, aggressive antiviral treatment was recommended for certain patient subgroups to reduce the severity and duration of symptoms. To assist States and other localities meet these needs, the U.S. Government distributed a quarter of the antiviral medications in the Strategic National Stockpile within weeks of the pandemic's start. However, there are no quantitative models guiding the geo-temporal distribution of the remainder of the Stockpile in relation to pandemic spread or severity. We present a tactical optimization model for distributing this stockpile for treatment of infected cases during the early stages of a pandemic like 2009 pH1N1, prior to the wide availability of a strain-specific vaccine. Our optimization method efficiently searches large sets of intervention strategies applied to a stochastic network model of pandemic influenza transmission within and among U.S. cities. The resulting optimized strategies depend on the transmissability of the virus and postulated rates of antiviral uptake and wastage (through misallocation or loss). Our results suggest that an aggressive community-based antiviral treatment strategy involving early, widespread, pro-rata distribution of antivirals to States can contribute to slowing the transmission of mildly transmissible strains, like pH1N1. For more highly transmissible strains, outcomes of antiviral use are more heavily impacted by choice of distribution intervals, quantities per shipment, and timing of shipments in relation to pandemic spread. This study supports previous modeling results suggesting that appropriate antiviral treatment may be an effective mitigation strategy during the early stages of future influenza pandemics, increasing the need for systematic efforts to optimize distribution strategies and provide tactical guidance for public health policy-makers.

## Introduction

In March/April 2009, a new swine-origin strain of influenza A/H1N1 virus (pH1N1) was detected in human populations in California and Mexico. The U.S. government declared a Public Health Emergency on April 26, 2009, followed on June 12 by a declaration of a global pandemic by the World Health Organization. By May 6, the U.S. Centers for Disease Control and Prevention (CDC) had distributed 11 million of the 50 million antiviral treatment courses held in the federal portion of the Strategic National Stockpile (SNS); since the recipients had local stockpiles as well, this allowed the CDC to exceed the pre-determined target of distribution of 31 million treatment courses of oseltamivir and zanamivir prior to the acceleration phase of the pandemic [1]. Accompanying the distribution was guidance recommending the use of antivirals primarily for treatment of suspected or confirmed cases of severe respiratory infection caused by this new strain [2]. Recent extrapolations from reported cases estimate that the pandemic caused over 50 million infections in the U.S. population; the majority of these have been asymptomatic or clinically mild, but pH1N1 nevertheless led to a substantial burden of hospitalization and death [3], [4].

In contrast to the clear guidance for public health leaders regarding the initial shipment of antivirals, the evidence base for determining the fate of the remainder of the stockpile is thin. Key policy statements have called for the use of mathematical models to support the development of an evidence-based policy for effectively deploying the remaining antiviral stockpile and other limited or costly measures to limit morbidity and mortality from pH1N1 [5], [6]. While mathematical modelers have taken great strides towards building predictive models of disease transmission dynamics within human populations, the computational complexity of these models often precludes systematic optimization of the demographic, spatial and temporal distribution of costly resources. Thus the typical approach has been to evaluate a relatively small set of candidate strategies [7]–[10].

Here, we use a new algorithm that efficiently searches large strategy spaces to analyze the optimal use of the U.S. antiviral stockpile against pandemic influenza prior to widespread and effective vaccination. Specifically, we seek to compute explicit release schedules for the SNS to minimize the cumulative infections in the first twelve months of an epidemic like that caused by pH1N1, with the objective of delaying disease transmission to allow for the development and deployment of a vaccine. We assume, in line with recent CDC guidance, that antivirals will be used exclusively for treatment of symptomatic individuals rather than wide-scale pre-exposure prophylaxis. We apply our algorithm to a U.S. national-scale network model of influenza transmission that is based on demographic and travel data from the U.S. Census Bureau and the Bureau of Transportation Statistics. We consider disease parameters estimated for the novel 2009 pH1N1 pandemic as well as more highly transmissible strains of pandemic influenza.

## Methods

We couple a fast, scalable, and adaptable optimization algorithm to a detailed simulation model of influenza transmission within and among the 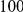 largest cities in the United States (total population of 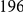 million). In brief, the method involves running a structured set of stochastic simulations of influenza transmission, with the optimization algorithm identifying the best choice of intervention policy based on a specific policy goal.

### Optimization method

**Figure 1:**
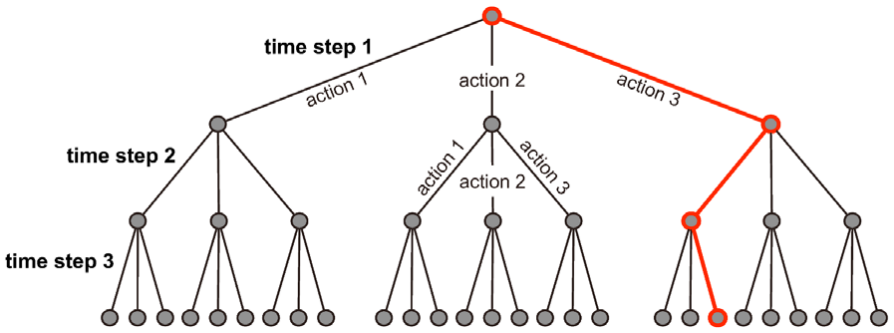
Simple Policy Tree. Suppose there are three possible actions and, in each time step, we can only choose one of them. Each level in the tree corresponds to a time step and branches represent possible actions. The red path through the tree represents the following three-step time-based intervention: First choose action 3, then action 1, and finally action 3 again. The policy tree for antiviral distribution has a similar organization. The UCT algorithm iteratively selects paths through the tree that represent intervention policies to be simulated.



A time-based intervention policy is a series of actions 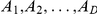 taken in sequence over 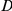 time periods (Fig. 1). Our objective is to rapidly search large sets of time-based intervention policies to find those that will be most effective at achieving a public health goal, such as limiting morbidity and mortality associated with influenza. Using a stochastic disease simulator, 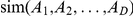, that evaluates the outcome of a given control strategy, we would like to solve the following optimization problem:

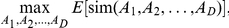
where 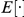 denotes the expectation with respect to the stochasticity in the simulator. For the optimization considered in this paper, the simulator returns the fraction of individuals not infected in the first 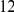 months of the epidemic.

To compute solutions to the above problem, we use trees to represent all possible policies (Fig. 1). The first (highest) level of a policy tree is a single node attached to several edges; each of those edges corresponds to one of the possible actions in the first time period and leads to a level-two node. Similarly each level-two node is attached to edges corresponding to all possible actions during the second time period, and so on. Each intervention policy corresponds to a unique path through the tree.

The naive approach to finding the optimal path through the tree is to simulate multiple disease outbreaks for each intervention policy (path) and record the expected morbidity or mortality (or other public health outcome measure). However, such exhaustive searches are computationally intractable for large trees. We can more efficiently search for the optimal policy by prudently sampling paths from the tree.

To strategically search the tree, we use an optimization algorithm called Upper Confidence Bounds Applied to Trees (UCT) [11], [12]. It selects paths from the tree using a multi-armed bandit algorithm inside of each tree node. The canonical application of a bandit algorithm is maximizing the total payoff from playing a set of slot machines for a fixed number of rounds, where the payoff distributions of the machines are unknown and, in each round, we may select only one machine. In this scenario, each edge emanating from the node corresponds to a slot machine that can be chosen by the node's bandit algorithm; for a policy tree, the edges correspond to possible policy actions. Before each policy simulation, bandit algorithms within the nodes select an edge to follow based on the results of prior trials. The combined choices of the bandit algorithms produce a path through the tree, corresponding to a sequence of public health actions, that is then passed into the simulation. The bandit algorithms determine which edge (action) to follow next by balancing two desirable characteristics: strong past performance and few prior trials. With this strategic path sampling, subtrees with good performance are explored more thoroughly than those with poor performance.

Specifically, suppose we are descending through the tree and have arrived at node 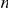 having 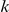 edges to the next level down, 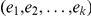, representing all possible subsequent actions. Let 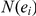 be the number of times we have used the intervention represented by edge 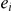 in prior simulations and 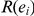 be a real number between 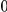 and 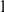, describing the average rewards observed during past simulations where 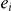 was chosen. In the analysis described below, the reward for a simulation is the fraction of individuals that remain uninfected during the outbreak. Now define 
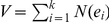
 to be the total number of times we have used descendants of 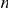 in past simulations. We then select the next edge as given by 
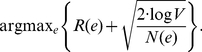
(1)


Initially, 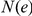 and 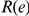 are set to zero for each edge. The first 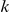 times we arrive at node 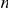, we choose the next edge uniformly randomly from the previously unsampled edges descending from the node, rather than choosing an edge based on Equation [1]. This gives initial estimates of 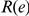 for each edge and guarantees that Equation [1] is well defined. At the end of each simulation run, if the simulation results in a reward of 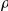, we update 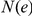 and 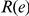 for each edge 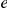 in the chosen policy path, as given by 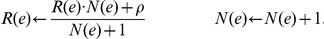



### Pandemic influenza transmission model

Our model includes the 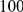 largest metropolitan areas in the United States, which we identified by aggregating Census Bureau Statistical Areas (CBSA) that share a common airport [13], [14]. We model movement among cities using both Census Bureau's County-To-County Worker Flow Files [15] and the Bureau of Transportation Statistics Origin and Destination Survey for all quarters of 2007, which contains a 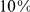 sample of all itineraries between U.S. cities [16]. We assume that each exposed or asymptomatic infectious traveler has some chance of starting an sustained epidemic in the destination city, by initiating a chain of transmission events to susceptible individuals. We assume further that this happens with probability 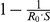, where 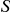 is the fraction of susceptible individuals in the destination city's population, as holds for a simple stochastic SIR model [17]. If there are 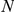 infected travelers this week from city 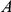 to city 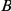 and the fraction of susceptibles in city 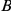 is 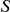, then the model draws a binomial random variable from the distribution 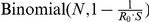 and creates that many new infected individuals in city 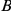. The number of infected travelers from city 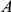 to city 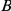 is calculated based on the travel data given as input, under the assumptions that symptomatic individuals do not travel and travelers are selected uniformly randomly from the population.

**Figure 2:**
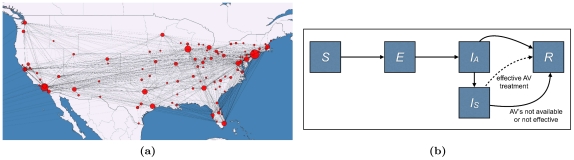
Disease Model. (2a) The U.S. network model for influenza transmission. Circle sizes represent numbers of inhabitants and line thickness represents the number of travelers between cities. (2b) Within-city compartmental model. The compartments are: susceptible (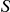), exposed (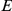), asymptomatic infectious (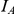), symptomatic infectious (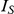), and resistant (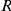). When infected individuals progress from asymptomatic to symptomatic they seek treatment at a rate 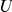 (uptake) and receive treatment if antiviral courses are available locally. While disease transmission is a continuous process, antivirals are distributed once per day to those requiring treatment. Antivirals are assumed to be 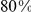 effective; and effectively treated cases immediately move to the recovered compartment. Untreated and ineffectively treated cases remain infectious until they recover naturally. The parameters of the compartmental model are described in Table 1.

Within each city, disease transmission is modeled using a compartmental model with five compartments: susceptible, exposed, asymptomatic infectious, symptomatic infectious, and recovered (Fig. 2b). Progression from one compartment to another is governed by published estimates for pandemic influenza transmission and disease progression rates, as given in Table 1. When infected individuals progress from asymptomatic to symptomatic they seek treatment at a rate 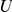 (uptake) and receive treatment if antiviral courses are available locally. While disease transmission is a continuous process, antivirals are distributed once per day to those requiring treatment. Antivirals are assumed to be 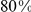 effective [18]–[24]; and effectively treated cases immediately move to the recovered compartment. Untreated and ineffectively treated cases remain infectious until they recover naturally. Epidemics are initialized assuming that there are 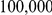 cases of pandemic flu in the United States (corresponding to the late June CDC estimate of over one million pH1N1 cases [25]) distributed stochastically, proportional to city sizes. Thus we are considering distribution policies that begin approximately one to two months following the initial emergence of the strain within the United States. Assuming that maximal flu vaccine coverage can be achieved within 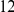 months of the onset of a pandemic, we terminate the simulations after 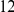 months or when all cases have recovered, whichever occurs first. Additional parameter values, initial conditions and time periods are explored in Supporting Information (Text S1, Video S1).

**Table 1:** Influenza transmission and intervention parameters.

Parameter	Symbol	Value	Reference
2009 pH1N1 Parameters			
reproductive number	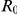	1.3, 1.4, 1.5	[28]–[31]
mean exposed period	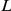	1 day	[29]
mean asymptomatic infectious period	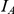	1.05	[29], [30]
mean total infectious period (asymptomatic + symptomatic)	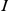	2.3 days	[29]
Pandemic Influenza Parameters			
reproductive number	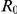	1.6, 2.0, 2.4	[10], [32], [33]
mean exposed period	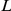	1.9 days	[10], [32], [33]
mean asymptomatic infectious period	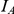	1.05	[10], [32], [33]
mean total infectious period (asymptomatic + symptomatic)	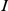	4.1 days	[10], [32], [33]
Intervention parameters			
antiviral efficacy	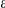	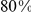	[18]–[24]
local stockpile half-life (wastage)	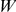	2 months	
antiviral uptake	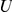	(0,1)	
mean infectious period prior to effective AV treatment		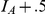 days	

The parameters for 2009 pH1N1 were calculated based on an incubation period of 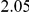 days, a serial interval of 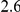 days, and an assumed exposed period of 1 day [29]. The calculations lead to an infectious period coinciding with infectivity that is within 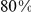 of peak levels [29]. The parameters for pandemic influenza are in agreement with the literature [10], [32], [33]. Note that, as depicted in Figure 2b, some infectious individuals may recover before becoming symptomatic.

### Antiviral policy actions

The model considers 11 possible antiviral stockpile actions every month over a twelve month period: distribution of 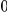, 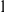, 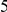, 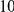, 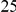 or 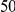 million courses apportioned either proportional to population or proportional to current prevalence. The total amount released during the twelve month period is not allowed to exceed the 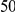 million courses available in the stockpile. We attempt to increase the realism of the model by assuming that, post distribution, unused courses “decay” through intra-jurisdictional misallocation (in the sense of inefficient matching of doses to cases) or frank loss at a rate 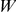. Wastage includes courses that are prescribed but go unused, are used to treat false positives, or are used too late in the course of disease to be effective. It is important to note that, for the purposes of this analysis, wastage does not refer to willful misuse. Clinically, we assume that antivirals are 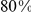 efficacious at reducing symptoms and forward transmission of the disease [18]–[24]. If an infected individual resides in a jurisdiction with remaining distributed antivirals, then they receive appropriate treatment (i. e., access to medications within 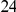 hours of onset of symptoms) with an uptake probability of 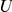. Effectively treated infected cases (i. e., 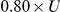) are immediately moved from the infectious to the recovered compartment, consistent with early evidence of rapid decline in viral titers in treated pH1N1 patients [26], [27]. Consistent with current CDC antiviral guidance, we did not model the use of antivirals for large scale prophylaxis of susceptible populations in the absence of infection.

### Computational requirements

Each optimization is based on 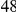 hours of computation on the Linux Lonestar Cluster at the Texas Advanced Computing Center, which offers a peak performance of 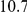 GFLOPS per optimization process. Roughly 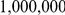 simulations can be done in this time; however, for this relatively small action space, the optimal policy is typically identified within six hours of computation.

## Results

**Figure 3:**
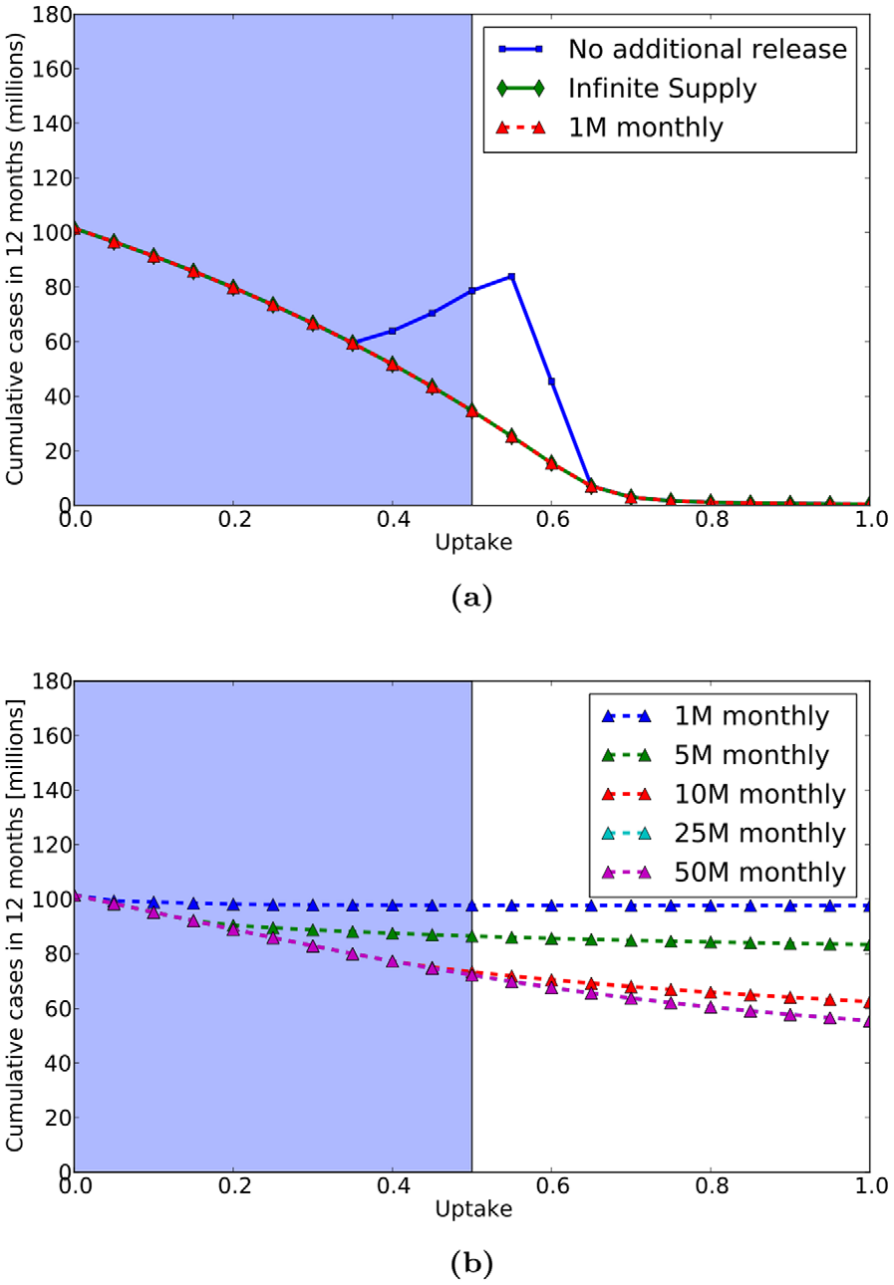
Antiviral SNS Policy Performance for 2009 pH1N1. Since the results all values of 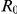 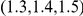 are similar, we only present those where 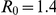. We compare the performance of various policies at different levels of antiviral uptake (horizontal axes) in terms of the cumulative number of cases in the first twelve months (vertical axes). (3a) Performance of antiviral control policies assuming a pre-distribution of 31 million courses proportional to population size. The infinite supply curve (green) corresponds to an idealized scenario where an infinite supply of antivirals is always available to each city. The no additional release curve (blue) assumes that no courses are released beyond the pre-distributed 31 million courses. The 1 M monthly curve (red) assumes that 1 million courses are distributed proportional to population size beginning in the third month of the pandemic, in addition to the 31 million pre-released courses. The infinite supply curve overlaps completely with the 1 M monthly curve. (3b) Performance of simple fixed releases proportional to population size assuming that no courses are pre-distributed and the first releases occur approximately three months into the US epidemic (two months after our simulations are initialized with 100,000 cases). In reality, antiviral uptake rates are limited by clinical manifestations of the disease (symptoms). For example, the presence of fever was one recommended criterion for prescribing antivirals for pH1N1. The blue highlighted regions of the curves indicate the range of uptake rates that might be medically feasible under proactive intervention, although the maximum attainable coverage for flu is possibly much lower than 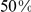.

First, we consider SNS distribution schedules for the 2009 pH1N1 pandemic (Figure 3). We found that simple distribution schedules such as releasing an arbitrary fixed quantity each month from the federal stockpile to the states proportional to population size perform optimally, due to the mild nature of the disease. In fact, we find very little difference between two extreme scenarios: (a) an infinite supply of antivirals available at all times in all cities, and (b) no federal stockpile releases beyond the 31 million initially purchased by states (Figure 3a). At low uptake, the initial 31 million courses are sufficient to meet demand; at high uptake, the aggressive early treatment essentially stops the epidemic before exhausting supplies; and only at intermediate levels is the the demand sufficiently high and the epidemic sufficiently long-lived to exhaust the available supplies (through a combination of treatment and wastage). A simple SNS release schedule of one million courses per month proportional to population size (in addition to the initial 31 million courses) is sufficient to meet the ongoing demand, regardless of uptake, and thus performs well as an infinite supply (Figure 3a).

The rapid allocation of the first Federal stockpile allotment and the contributions of antivirals by the states to provide for the 31 million courses in the early stages of the epidemic are critical in these simulations. If we remove these courses and assume conservatively that the first Federal distributions take place approximately 3–4 months into the pandemic, we find that antiviral treatment only modestly slow transmission (Figure 3b). Simple release schedules are predicted to perform much more poorly without the initial distribution, with large early distributions outperforming regular small distributions.

**Figure 4:**
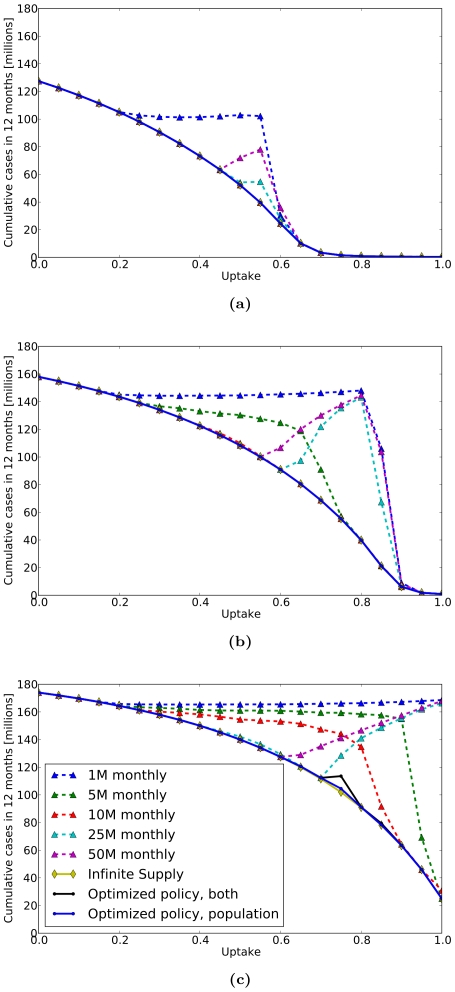
Antiviral SNS Policy Performance for Pandemic Influenza. We compare the performance of various policies at different levels of antiviral uptake (horizontal axes) in terms of the cumulative number of cases in the first twelve months (vertical axes) for pandemic strains with reproduction numbers: (4a) 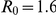, (4b) 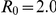, and (4c) 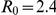. Each figure displays compares several policies including: 1) several fixed monthly distributions proportional to population size, 2) an idealized scenario with infinite supply of antivirals available to each city, and 3) optimized policies allowing either a combination of population and prevelance-based releases or solely population based releases. For strains with 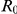 of 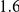 and 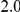, some simple policies are predicted to perform as well as the idealized scenario, however, for 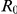 of 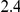, no simple policies are comparable with the idealized scenario. The optimized control policies always outperform the simple policies and typically match the performance of the idealized scenario. Similar to the results shown in Figure 3, the true maximal attainable uptake rates would be limited by the clinical symptoms of a future pandemic.

We assumed a reproduction number of 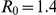 for 2009 pH1N1 [28]–[31]. In contrast, we obtained different results for more transmissible strains of pandemic influenza, with reproduction numbers 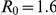, 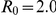, and 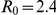 [10], [32], [33]. Figures 4a–4c include the following performance curves:

1. Several policies in which the stockpile is released monthly in fixed quantities proportional to population size, until the 12 month time horizon is reached or until the SNS is depleted. The releases range from 1 million courses for 12 months, to a single release of 50 million courses.
2. An idealized scenario with an infinite supply of antivirals available to each city throughout the epidemic. The outcome of this scenario indicates the maximal potential impact of antiviral use at any given utilization rate, free of any logistical constraints on supply.
3. Two optimized strategies resulting from our analysis. In one optimized strategy, we allowed releases to be either proportional to population size or proportional to influenza prevalence in the city. In the other optimized strategy, we allowed only releases proportional to population.

Unlike the 2009 pH1N1 scenario, more contagious strains of pandemic influenza require greater care in selection of antiviral release strategy. For example, the simple policy of 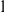 million courses released monthly now significantly under performs the other strategies (Figure 4). Optimized release policies (computed by UCT optimization) consistently perform almost as well as the infinite supply scenario. In all cases, except when the reproduction number is 2.4 and the uptake is 0.75, the *optimality gap* – difference in performance of computed policy and best idealized outcome divided by the best idealized outcome – is at most one tenth of one percent. For the single outlier scenario, the optimality gap is 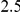
% for a policy using population only releases and 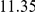
% for a policy allowing both population and prevalence releases. The under performance of the mixed strategy stems from the vast size and structure of the policy tree over which optimization is performed, and is discussed further below.

Under realistic assumptions about transmissability, we found that simple release schedules perform almost as well as the optimized policies. For example, at 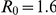, the policies of releasing 5 million or 10 million courses monthly performed as well as the infinite supply scenario; at 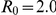, only the policy of 10 million courses released monthly performed as well as the infinite supply scenario. However, at the extreme range of influenza transmissability (i.e., 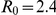), none of the simple policies performed as well as the optimized policies.

In all of the simulations, the proportion of infected individuals who seek timely treatment (what we refer to as uptake) has a dramatic impact on both policy optimality and outcomes.

**Figure 5:**
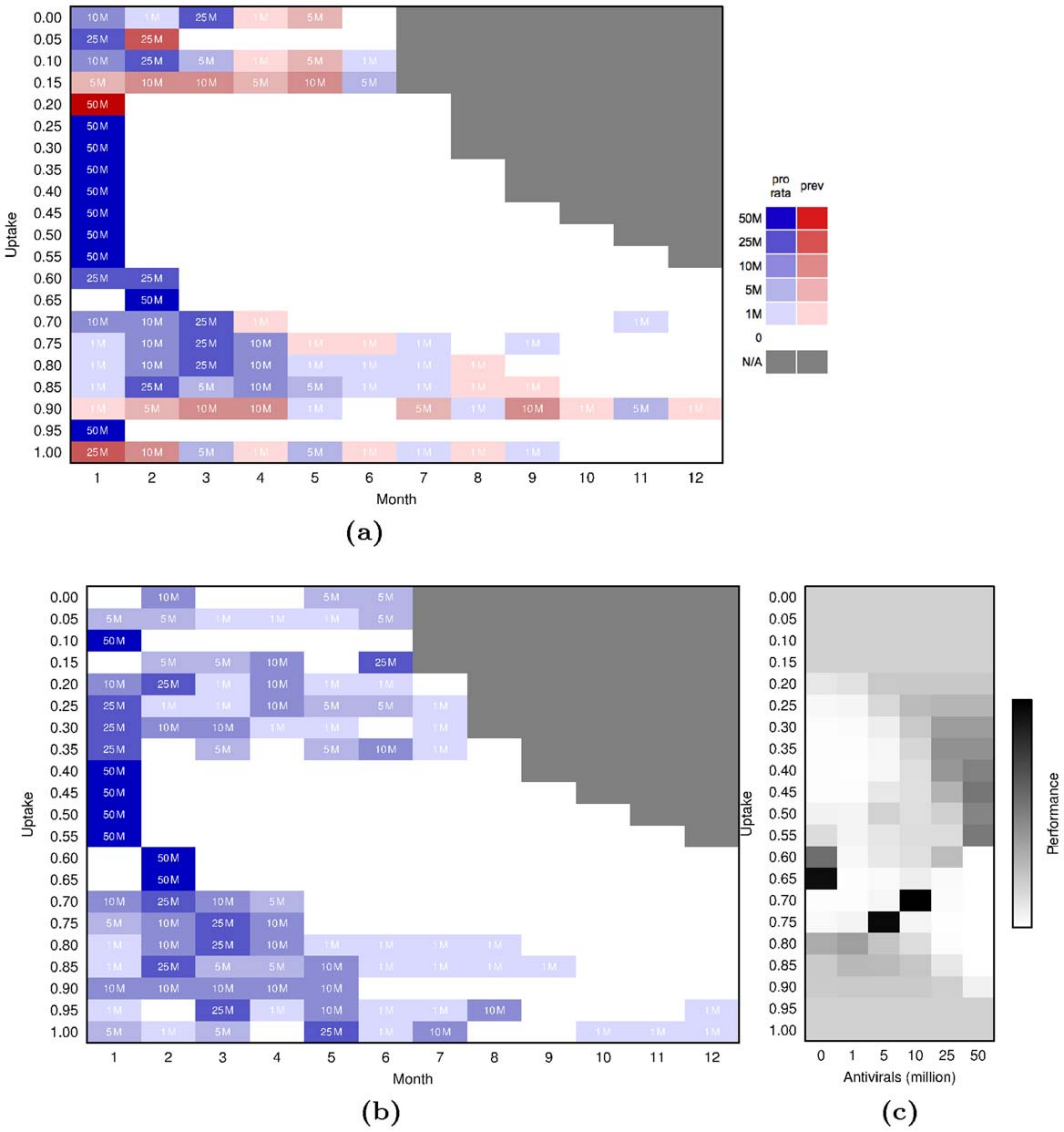
Optimized Policies for Pandemic Influenza with a Reproduction Number of 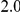. (5a) Optimized policies combining prevalence-based (red) and population-based distributions (blue). Each row gives the optimized sequence of actions for a given value of uptake. (5b) Optimized policies allowing only population-based distributions. (5c) Performance of possible actions for the first distribution for a population-based policy, two months after the pandemic has reached 100,000 cases. Shading indicates number of times an action was visited during the optimization routine, and is thus proportional to the performance of the action.

Figure 5 shows the optimized policies for pandemic flu with a reproduction number of 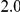, allowing distributions either proportional to population size or proportional to local disease prevalence (Figure 5a) or only proportional to population size (Figure 5b). At an uptake of 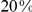, the optimized mixed strategy entails a single distribution of 50 M courses proportional to local prevalence, while the optimized population-based strategy distributes 10 million courses in the first month, followed by 25 million, 1 million, 10 million, 1 million, and 1 million in the following months. However, Figure 4b shows that these two optimized strategies as well as fixed releases of 5 million to 50 million per month proportional to population size perform optimally, on par with the infinite supply scenario. Figure 5c furthermore illustrates that, at uptake of 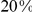, an initial release of 25 or 50 million courses proportional to population size performs as well as the (population-only) optimized policy of 10 million courses. Although there are often multiple optimal policies, this is not the case for uptake rates ranging between 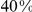 and 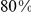. At these relatively high levels of treatment, the optimized policy is more clearly defined (Figure 5c) and most simple policies perform suboptimally (Figure 4b).

Even when prevalence-based releases are allowed, the optimal policies tend to be dominated by population-based releases (Figure 5a). This combined with the comparable performance of exclusively population-based policies across all scenarios suggests that prevalence-based distributions are probably unnecessary. Thus we focus on Figures 5b and 5c to gain quantitative insights into the relationship between uptake and best policy. At low levels of uptake (between 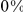 and 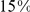) essentially all releases perform optimally (Fig. 5c). At these levels of uptake, so few people are treated that the initial 31 M courses satisfies the demand. For uptakes between 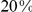 and 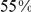, additional courses from the Federal SNS are necessary to meet demand, and thus the optimal policies involve sizable early releases. For uptakes between 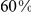 and 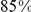, wastage becomes even more of an issue and thus the success of the policy is highly sensitive to the exact distribution of the initial releases. Here, the optimal schedules delay and extend the distribution over several months (Fig. 5b). Finally, for the highest levels of uptake (greater than 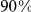), the pre-released 31 M courses are sufficient to control the epidemic, as seen also in Figure 4b.

## Discussion

Since avian influenza H5N1 became a potential public health threat in 2003, public health agencies around the globe have been planning for the next influenza pandemic. While the concerted response to pH1N1 reflects this careful preparation, several expected and unexpected events, including its apparent North American origin, the rapid overburdening of U.S. laboratory capacity, non-uniform testing and treatment policies among U.S. states, and delays in production of a viable vaccine, all reinforce the need for a dynamic and quantitative playbook for pandemic mitigation using pharmaceutical countermeasures.

By adapting an established algorithm to optimize disease mitigation policies, this study provides an advance from the traditional candidate strategy approach to rapid and systematic analysis of numerous policy options. This is just one of many possible optimization methods suitable for this purpose [34]–[37]. Our choice of UCT was based on the insight that, with some careful modeling, disease intervention strategies can be nicely mapped onto policy trees and that this approach can be coupled to any stochastic epidemic model. This approach has performed successfully on large policy trees [38] and has favorable convergence properties [35]. In particular, unlike simulated annealing and genetic algorithms, it is guaranteed to eventually converge on the optimal policy.

The UCT algorithm preferentially samples subtrees of the policy tree that have performed well in the past (see [35] for a mathematical discussion). The algorithm performs best when all of the policies within a single subtree of the policy tree perform similarly; it can then effectively determining the “goodness” of any subtree by sampling it only a few times. To achieve algorithmic efficiency, one should therefore use expert knowledge and intuition to structure the policy tree in this way. If there is a single optimal solution in a subtree surrounded by many poorly performing solutions, then the UCT algorithm may require many simulations to find it (although it is guaranteed to eventually do so). Unbalanced policy trees, with one subtree much deeper than another, are natural topologies to produce such an unfavorable grouping of solutions. The single outlier in Figure 4c was likely caused by a combination of imbalance in the antiviral policy tree and the sheer volume of policy options at each time point. First, one subtree includes releasing the entire SNS in the first month with no actions following, while another involves waiting several months to release a small sequence of antivirals. Second, allowing both population-based and prevalence-based distributions increases the options available at each time point and reduces the depth to which policies can be optimized in a given amount of time. Although we know that the outlier is not the true optimal solution, we have have opted to present it in the graphs to highlight intuition on the algorithm's performance. For UCT, additional simulations are guaranteed to improve the optimality gap; in this case, they would have moved the optimized mixed policy to at least match the optimized population-based policy.

We initially conducted this analysis during summer 2009, as the pH1N1 pandemic was unfolding, in response to questions posed to us by public health agencies regarding the effective use of antivirals prior to the availability of pH1N1 vaccines. Although the CDC has since issued antiviral guidelines and pH1N1 vaccines are now widely available, our analyses provide insight into the likely impacts of antivirals on pH1N1 transmission to date and effective strategies for antiviral-based mitigation of future flu pandemics.

Our analysis suggests that while pH1N1 may have been slowed with targeted, aggressive, and clinically successful use of antivirals, the impact of such a policy would have been highly insensitive to the choice of Federal distribution schedule. The 31 million courses already available to states prior to the pandemic would have gone a long way towards meeting the early demand. However, for more contagious pandemic strains (with higher reproduction numbers), use of an optimized distribution schedule would be expected to significantly improve the intervention outcome. In some cases, simple strategies involving regular fixed releases perform as well as more complex optimized strategies. For example, for a pandemic strain with 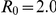, a monthly distribution of 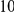 million regimens divided proportional to population among the states consistently matches or outperforms other policy options, regardless of the levels of uptake or potential misallocation or loss of medication, which are likely in a complex health emergency response setting. Slight variations on this policy, for example, regular distributions of 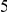 or 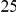 million courses are predicted to perform significantly worse across a large range of uptake values. From a public health perspective, the best policies are those that have robust performance in a variety of scenarios. The search for such robust policies can be implemented directly into the optimization method, by having the simulator sample from a prior distribution of scenarios. However, no guarantee exists that a single policy can be robust against all the scenarios under consideration.

Our optimization allowed for the possibility of distributions proportional to prevalence, although such actions are not consistent with the current CDC policy and would likely be both politically and logistically difficult. Technically, implementing such a scheme would impose a major surveillance burden, as it would necessitate the estimation of prevalence rates throughout the nation based on noisy or delayed data. Notably, the results suggest that prevalence-based distributions are not expected to enhance the impact of antiviral treatments.

The impact of antiviral treatment policies is naturally sensitive to the rate at which individuals who should receive these countermeasures actually do in clinical settings (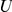). From a study of pH1N1 antiviral uptake in Milwaukee during summer 2009, preliminary estimates of the fraction of reported cases receiving treatment within 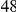 hours of developing symptoms are less than 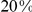 [39]. In September 2009, the CDC issued antiviral guidelines the encouraged prioritization of high risk cases and discouraged antiviral treatment of typical cases. This suggests that throughout the summer and fall of 2009, we have likely been in the range where all strategies perform equally poorly and are predicted to minimally mitigate transmission. This is not to say that antivirals have had no impact on pH1N1 outcomes: to date, they have been used to significantly reduce morbidity and mortality associated with pH1N1 when used in potentially severe cases. Thus, for future pandemics, public health measures to increase the rates of antiviral usage beyond current levels may have the potential to slow transmission prior to the availability of vaccines. An increase in uptake rates may be practically limited by clinical symptoms of the disease in question, such as the presence of fever, which was one recommended criterion for prescribing antivirals. Our analysis shows, however, that the impact of antiviral control measures depends not only on the rates of uptake but also may critically depend on the Strategic National Stockpile distribution schedule used to sustain that uptake, particularly for highly contagious strains.

We did not consider the development of antiviral resistance in this study. Currently circulating strains of seasonal influenza have acquired resistance to oseltamivir [40] and there is evidence that the pH1N1 virus is capable of experiencing genetic mutations that confer resistance to at least one neuraminidase drug; thankfully, to date there is little evidence of sustained transmission of such mutations. We also did not incorporate the use of antivirals for prophylaxis, the future availability of vaccines, simultaneous use of vaccines or NPI's like school or event closures, or the option of targeting the stockpile towards particular demographic groups, all of which are likely important and may influence the optimal policy.

The effectiveness of any antiviral policy will depend critically on the extent to which antivirals reduce the severity and transmission of flu. Our assumptions regarding antiviral efficacy are in agreement with the literature [18]–[24]; most of these studies assume maximum likelihood-based estimates of antiviral efficacies calculated by Longini et. al [32] using data from a clinical study by Welliver et. al [41]. More recent clinical trials indicate that the odds of a secondary infection in individual contacts decreases by approximately 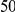
% when antivirals are used on the day of onset (OR: 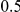, 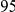
% CI: 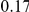, 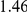) [42], [43]. While the antiviral efficacy we assumed here lies well within the confidence intervals estimated in these papers, better estimates of these and other parameter values will certainly improve the future optimization studies.

In this study, we assume that all distributed antiviral courses undergo wastage. There are multiple potential causes of wastage, including courses that are prescribed to patients who never use them, use them to treat diseases other than flu, or use them too late in their flu infection to significantly impact transmission. Since there is very little information on the rates at which such loss or misuse occurs, let alone how these rates change over the course of a pandemic, we have modeled wastage using a generic decay function. Comparisons between the optimized policies (assuming wastage) and an infinite supply scenario (with no wastage) suggest that there exist distributions schedules that effectively avert potential public health costs associated with wastage. Although better estimates of the magnitude and dynamics of wastage would improve the accuracy of the model and may suggest slightly different optimal strategies, we expect that those strategies will still overcome the potential detrimental effects of wastage.

Our work complements a growing body of modeling studies on the distribution and timing of antiviral and vaccination policies. Bajardi et al. recently developed a similar large-scale geographic disease spread model, with which they showed that a vaccination campaign following the initial outbreak may require additional mitigation strategies to delay the epidemic [44]. Danon et al. showed, however, that such models can be sensitive to the addition of movement patterns not captured in census data; specifically, the addition of random movement can hasten an epidemic [45]. A modeling study by Handel et al. suggests that when antivirals are the only mode of control, using antivirals towards the end of the epidemic to minimize overshoot is a good control policy [46]. An intuitive mathematical model developed by Lipstich et al. shows that while antiviral use likely promotes the rise of antiviral resistant strains, they nonetheless can significantly delay the epidemic [47]. Studies by Nuño et al. and Wu et al. also suggest that antivirals used for treatment can slow the spread of the epidemic [48], [49]. Vaccination studies may provide some insight into the potential impacts of large scale antiviral prophylaxis, which we have not considered in our analysis. For example, using a deterministic meta-population model, Wu et al. showed that it may be preferable to allocate large quantities of vaccines to particular geographic areas in order to achieve local herd immunity as opposed to distributing vaccines proportional to population [50]. Ball et al. have studied a related vaccine distribution problem on a graph-based model of disease spread, and also show that targeting local groups performs well if the entire sub-population can be effectively protected [51]. Finally, Bootsma and Ferguson studied the 1918 influenza pandemic, and found that the timing of interventions can be critical, with delays in implementation and premature lifting of interventions reducing the impact of control measures [52].

From rapid genetic sequence analysis to automated syndromic surveillance systems, public health emergency response is rapidly improving in technical capabilities both in the U.S. and worldwide; the rapid response to and characterization of the novel pandemic influenza A (pH1N1) virus is a testament to this. However, planning the policies of public health response to such identified and emergent threats remains a highly non-quantitative endeavor. We present here a policy optimization approach that is highly modular and can be easily adapted to address multiple additional issues. Our hope is that these quantitative methods will assist clinical experts in developing effective policies to mitigate influenza pan- and epidemics using a combined arsenal of vaccines, antivirals and non-pharmaceutical interventions. Specifically, a very similar analysis can be used at the international level to optimize global allocation of the WHO's limited antiviral stockpile to resource-poor countries. One can substitute any stochastic model of disease transmission, at any scale, for our national-scale, U.S. influenza model. In addition, while the optimization algorithm is particularly well suited for time-based interventions, any well-behaved policy space can be used [35]. The approach should thereby facilitate a more comprehensive consideration of pandemic policy options, and will perhaps confirm the efficacy of the current policy or suggest more promising strategic options for the future.

## Supporting Information

Text S1 Supplemental information, including detailed model description, model validation runs, and additional optimization scenarios.
(PDF)

Video S1 Supplemental visualizations for scenarios described in Text S1.
(MP4)

## References

[1] US Centers for Disease Control (2009). CDC health update: Swine influenza A (H1N1) update: New interim recommendations and guidance for health directors about strategic national stockpile material.. http://www.cdc.gov/h1n1u/HAN/042609.htm.

[2] US Centers for Disease Control (2009). Questions & answers: Antiviral drugs, 2009–2010 flu season.. http://www.cdc.gov/h1n1flu/antiviral.htm.

[3] US Centers for Disease Control (2009). CDC estimates of 2009 H1N1 influenza cases, hospitalizations and deaths in the United States, April 2009-January 16, 2010.. http://cdc.gov/h1n1flu/estimates/April.

[4] Reed C, Angulo FJ, Swerdlow DL, Lipsitch M, Meltzer MI (2009). Estimates of the prevalence of pandemic (H1N1) 2009, united states, April–July 2009.. Emerging Infectious Diseases.

[5] White House National Security Staff (2009). National framework for 2009-H1N1 influenza preparedness and response..

[6] Department of Homeland Security (2007). Homeland security presidential directive 21.. http://www.dhs.gov/xabout/laws/gc.

[7] Longini I, Halloran M, Nizam A, Yang Y (2004). Containing pandemic influenza with antiviral agents.. American Journal of Epidemiology.

[8] Ferguson N, Cummings D, Cauchemez S, Fraser C, Riley S (2005). Strategies for containing an emerging influenza pandemic in Southeast Asia.. Nature.

[9] Bansal S, Pourbohloul B, Meyers L (2006). A comparative analysis of influenza vaccination programs.. PLoS Medicine.

[10] Germann TC, Kadau K, Longini IM, Macken CA (2006). Mitigation strategies for pandemic influenza in the United States..

[11] Kocsis L, Szepesvári C (2006). Bandit based monte-carlo planning.. Machine Learning: ECML.

[12] Auer P, Cesa-Bianchi N, Fischer P (2002). Finite-time analysis of the multiarmed bandit problem.. Machine Learning.

[13] Bureau of Transportation Statistics (2008). Airport master coordinate data.. http://www.transtats.bts.gov/Fields.asp?Table.

[14] Census Bureau (2000). About metropolitan and micropolitan statistical areas.. http://www.census.gov/population/www/metroareas/aboutmetro.html.

[15] Census Bureau (2000). County-to-county worker flow files.. http://www.census.gov/population/www/cen2000/commuting/index.html.

[16] Bureau of Transportation Statistics (2007). Origin and destination survey, all quarters of 2007.. http://www.transtats.bts.gov/Fields.asp?Table.

[17] Keeling MJ, Rohani P (2008). Modeling Infectious Diseases..

[18] Lee V, Chen M (2007). Effectiveness of neuraminidase inhibitors for preventing staff absenteeism during pandemic influenza.. Emerging Infectious Diseases.

[19] McCaw J, McVernon J (2007). Prophylaxis or treatment? Optimal use of an antiviral stockpile during an influenza pandemic.. Mathematical Biosciences.

[20] Lee V, Phua K, Chen M, Chow A, Stefan M (2006). Economics of neuraminidase inhibitor stockpiling for pandemic influenza, Singapore.. Emerging Infectious Diseases.

[21] Doyle A, Bonmarin I, Levy-Bruhl D, Strat Y, Desenclos JC (2006). Influenza pandemic preparedness in France: modelling the impact of interventions.. Journal of Epidemiology and Community Health.

[22] Barnes B, Glass K, Becker N (2007). The role of health care workers and antiviral drugs in the control of pandemic influenza.. Mathematical Biosciences.

[23] Hota S, McGeer A (2007). Antivirals and the control of influenza outbreaks.. Clinical Infectious Diseases.

[24] Lipsitch M, Cohen T, Murray M, Levin B (2007). Antiviral resistance and the control of pandemic influenza.. PLoS Medicine.

[25] Schuchat A (2009). CDC telebriefing on investigation of human cases of novel influenza A (H1N1).. http://www.cdc.gov/media/transcripts/2009/t090626.htm.

[26] Ling LM, Chow AL, Lye DC, Tan AS, Krishnan P (2010). Effects of Early Oseltamivir Therapy on Viral Shedding in 2009 Pandemic Influenza A (H1N1) Virus Infection.. Clinical Infectious Diseases.

[27] Yu H, Liao Q, Yuan Y, Zhou L, Xiang N (2010). Effectiveness of oseltamivir on disease progression and viral RNA shedding in patients with mild pandemic 2009 influenza A H1N1: opportunistic retrospective study of medical charts in China.. British Medical Journal.

[28] Fraser C, Donnelly CA, Cauchemez S, Hanage WP, Van Kerkhove MD (2009). Pandemic potential of a strain of influenza A (H1N1): Early findings.. Science.

[29] Ghani AC, Baguelin M, Griffin J, Flasche S, Pebody R, van Hoek AJ (2009). The early transmission dynamics of H1N1pdm influenza in the United Kingdom..

[30] Pourbohloul B, Ahued A, Davoudi B, Meza R, Meyers L (2009). Initial human transmission dynamics of a novel swine-origin influenza A (H1N1) virus (S-OIV) in North America.. Influenza and Other Respiratory Viruses.

[31] Yang Y, Sugimoto JD, Halloran ME, Basta NE, Chao DL (2009). The transmissibility and control of pandemic influenza A (H1N1) virus.. Science.

[32] Longini IM, Halloran ME, Nizam A, Yang Y (2004). Containing pandemic influenza with antiviral agents.. American Journal of Epidemiology.

[33] Mills CE, Robins JM, Lipsitch M (2004). Transmissibility of 1918 pandemic influenza.. Nature.

[34] Azadivar F (1999). Simulation optimization methodologies..

[35] Coquelin P, Munos R (2007). Bandit algorithms for tree search.. Computing Research Repository:abs/cs/0703062.

[36] Mannor S, Tsitsiklis J (2004). The sample complexity of exploration in the multi-armed bandit problem.. Journal of Machine Learning Research.

[37] Dar E, Mannor S, Mansour Y (2002). PAC bounds for multi-armed bandit and Markov decision processes..

[38] Gelly S, Wang Y (2006). Exploration exploitation in Go: UCT for Monte-Carlo Go.. 20th annual Conference on Neural Information Processing Systems.

[39] Goldstein E, Cowling B, O'Hagan J, Danon L, Fang V (2010). Oseltamivir for treatment and prevention of pandemic influenza A/H1N1 virus infection in households, Milwaukee, 2009.. BMC Infectious Diseases.

[40] Hurt A, Ernest J, Deng Y, Iannello P, Besselaar T (2009). Emergence and spread of oseltamivir-resistant A(H1N1) influenza viruses in oceania, south east asia and south africa.. Antiviral Research.

[41] Welliver R, Monto AS, Carewicz O, Schatteman E, Hassman M (2001). Effectiveness of Oseltamivir in Preventing Influenza in Household Contacts: A Randomized Controlled Trial.. The Journal of the American Medical Association.

[42] Goldstein E, Cowling B, O'Hagan J, Danon L, Fang V (2010). Oseltamivir for treatment and prevention of pandemic influenza A/H1N1 virus infection in households, Milwaukee, 2009.. BMC Infectious Diseases.

[43] Ng S, Cowling B, Fang V, Chan K, Ip D (2010). Effects of oseltamivir treatment on duration of clinical illness and viral shedding and household transmission of influenza virus.. Clinical Infectious Diseases.

[44] Bajardi P, Poletto C, Balcan D, Hu H, Goncalves B (2009). Modeling vaccination campaigns and the fall/winter 2009 activity of the new A(H1N1) influenza in the Northern hemisphere.. Emerging Health Threats Journal.

[45] Danon L, House T, Keeling MJ (2009). The role of routine versus random movements on the spread of disease in Great Britain.. Epidemics.

[46] Handel A, Longini IM, Antia R (2007). What is the best control strategy for multiple infectious disease outbreaks?. Proceedings of the Royal Society B: Biological Sciences.

[47] Lipsitch M, Cohen T, Murray M, Levin BR (2007). Antiviral resistance and the control of pandemic influenza.. PLoS Medicine.

[48] Nuño M, Chowell G, Gumel AB (2007). Assessing the role of basic control measures, antivirals and vaccine in curtailing pandemic influenza: scenarios for the US, UK and the Netherlands.. Journal of The Royal Society Interface.

[49] Wu JT, Leung GM, Lipsitch M, Cooper BS, Riley S (2009). Hedging against antiviral resistance during the next influenza pandemic using small stockpiles of an alternative chemotherapy.. PLoS Medicine.

[50] Wu JT, Riley S, Leung GM (2007). Spatial considerations for the allocation of pre-pandemic influenza vaccination in the United States.. Proceedings of the Royal Society B: Biological Sciences.

[51] Ball F, Mollison D, Scalia-Tomba G (1997). Epidemics with two levels of mixing.. The Annals of Applied Probability.

[52] Bootsma MCJ, Ferguson NM (2007). The effect of public health measures on the 1918 influenza pandemic in U.S. cities.. Proceedings of the National Academy of Sciences.

