# Correction: Optimizing Tactics for Use of the U.S. Antiviral Strategic National Stockpile for Pandemic Influenza

**DOI:** 10.1371/annotation/cf0572ee-8088-4bcc-8680-e8ebb4f05ce3

**Published:** 2011-03-24

**Authors:** Nedialko B. Dimitrov, Sebastian Goll, Nathaniel Hupert, Babak Pourbohloul, Lauren Ancel Meyers

One of the author's funding sources was not acknowledged in the published manuscript. The authors wish to add the following funding information to the Funding section: "This work was supported by grants to LM from NIH Models of Infectious Disease Agent Study (MIDAS) (U01-GM087719-01), the James S. McDonnell Foundation, and NSF (DEB-0749097) and grants to BP from CIHR(PTL-97125 and PAP-93425) and the Michael Smith Foundation for Health Research."

