# Supplementary material for: Optimizing Tactics for Use of the U.S. Antiviral Strategic National Stockpile for Pandemic Influenza
Source: PLoS One. 2011 Jan 19;6(1):e16094. doi: 10.1371/journal.pone.0016094 (PMC3023704; doi:10.1371/journal.pone.0016094)
Supplement: Text S1 — Supplemental information, including detailed model description, model validation runs, and additional optimization scenarios. (PDF) [file pone.0016094.s001.pdf]

Supplemental Analysis for Optimizing Tactics for use of the U.S. Antiviral Strategic National Stockpile for Pandemic (H1N1) Influenza, 2009

Nedialko B. Dimitrov, Sebastian Goll, Nathaniel Hupert,
Babak Pourbohloul, Lauren Ancel Meyers

May 19, 2010

1 Simulation

We simulate the spread of disease within and between the 100 largest metropolitan areas in the United States. Disease is transmitted within cities according to a deterministic compartmental SEIR model (*city model*). Disease moves stochastically between cities via infected travelers (*network model*). In each *simulation run* we alternately simulate a week (i. e., 7 days) of disease transmission within each city using the city model and then simulate between-city transmission using the network model, as described below. This process is repeated for 53 weeks or until the disease dies out, whichever occurs first.

1.1 City Model

Let $\mathbb{C}$ be the *set of all cities*. For each city $i \in \mathbb{C}$ we assume a deterministic SEIR model with 5 compartments:

1. *susceptible* (S_i)
2. *latent* (E_i)
3. *asymptomatic infectious* (I_i^{A})
4. *symptomatic infectious* (I_i^{S})
5. *recovered* (R_i).

These compartments are connected by the following differential equations, and include parameters for *transmission rate* (β), *mean latent period* ($1/\nu$), *mean asymptomatic period* ($1/\mu$), and *mean infectious period* ($1/\gamma$) of the disease.

$$\begin{aligned}
\mathrm{d}S_i/\mathrm{d}t &= -\beta(I_i^{\mathrm{A}} + I_i^{\mathrm{S}}) \cdot S_i, \\
\mathrm{d}E_i/\mathrm{d}t &= \beta(I_i^{\mathrm{A}} + I_i^{\mathrm{S}}) \cdot S_i - \nu E_i, \\
\mathrm{d}I_i^{\mathrm{A}}/\mathrm{d}t &= \nu E_i - (\mu + \gamma) \cdot I_i^{\mathrm{A}}, \\
\mathrm{d}I_i^{\mathrm{S}}/\mathrm{d}t &= \mu I_i^{\mathrm{A}} - \gamma I_i^{\mathrm{S}}, \\
\mathrm{d}R_i/\mathrm{d}t &= \gamma(I_i^{\mathrm{A}} + I_i^{\mathrm{S}}).
\end{aligned}$$

Individuals who seek antiviral treatment do so only once, within 24 hours of progressing from asymptomatic to symptomatic, as described below in Section 1.4. In addition to the five compartments, the model tracks the number of individuals treated with antivirals both effectively and ineffectively.

An epidemic within a city i is assumed to terminate when there is the equivalent of less than half of an individual anywhere in the latent, asymptomatic, or infectious compartments.

1.2 Network Model

Between-city disease transmission occurs at the end of each simulated week. We assume that infectious individuals showing symptoms do not travel; and that each latent or asymptomatic infected traveler sparks a deterministic epidemic in the destination city (starting with a single transmission event) with probability $p_{\mathrm{I}} = 1 - \frac{1}{R_0 \cdot S_j}$ [10].

Let the population size of city i be denoted as $\mathrm{size}_{(i)}$. The total number of infected individuals eligible for travel in city i is $\mathrm{size}_{(i)} \cdot (E_i + I_i^{\mathrm{A}})$. Let $\mathrm{flux}_{(i \to j)}$ denote the daily number of travelers from city i to city j. Then each infected individual in city i has a probability of $\frac{\mathrm{flux}_{(i \to j)}}{\mathrm{size}_{(i)}}$ to travel to city j each day. The probability of traveling to city j at any point during the week is then $p_{i \to j}^{\mathrm{travel}} = (1 - (1 - \frac{\mathrm{flux}_{(i \to j)}}{\mathrm{size}_{(i)}})^7)$. Thus, the expected number of infected travelers from city i to city j is $\tau_{ij} = p_{i \to j}^{\mathrm{travel}} \cdot \mathrm{size}_{(i)} \cdot (E_i + I_i^{\mathrm{A}})$.

To determine the number of new infections transmitted by infected travelers from city i to city j, we draw a binomial random variable with number of trials equal to τ_{ij}, rounded to the nearest integer, and probability of success equal to p_{I}. This transmission process is repeated for each pair of cities at the end of each week.

1.3 Initial Condition

At the start of each simulation, we specify the number of initial infected individuals for each city, each of whom start out in the latent compartment.

1.4 Intervention

We model two types of antiviral distributions. *Pro rata* distributions are provided to cities proportional to population sizes; and *prevalence-based* distributions are provided to cities proportional to current prevalences of H1N1, where the prevalence is the total number of symptomatic individuals in the city at the time (those in the I^{S} compartment). Latent and asymptomatic individuals are not included, since it is not feasible to identify those individuals in a real situation.

The administration of antivirals in the simulation is dependent on two parameters, *uptake* denoted as U and *wastage* denoted as W. Uptake is the fraction of symptomatic infectious individuals that seek treatment. Wastage is the length of time required to diminish the released and available antivirals in a city, through misuse or loss, to half of their original size.

Antiviral treatment is modeled as follows. Each city i has a cache of available antivirals. Antivirals are administered at the end of each day in the simulation. Only individuals that have progressed from I_i^{A} to I_i^{S} within the prior 24 hours seek antiviral treatment, and do so with probability U. Symptomatic individuals are assumed not to seek antivirals beyond the first 24 hours. Although, in reality, H1N1 patients often receive antivirals at later stages, we assume that late-stage treatment does not significantly reduce transmission, and incorporate late-stage use of antivirals into the wastage parameter W rather than modeling it explicitly. The antivirals are assumed to be 80 % effective. Individuals effectively treated with antivirals are moved to the R_i

compartment. Individuals that do not seek antivirals and individuals for whom antivirals are administered but are not effective remain in the I_i^{S} compartment until recovering naturally. The city's antiviral cache is decreased by the number of antiviral courses sought that day (or by the number of remaining regimens if there are fewer available than sought). Following treatment, the local cache is decreased multiplicatively to model wastage, with the amount of decrease determined by the half-life wastage parameter W.

2 Data

2.1 Network Data

The cities in our model are based on the Census Bureau's Core Based Statistical Areas (CBSA). Roughly, each CBSA is "a core area containing a substantial population nucleus, together with adjacent communities having a high degree of economic and social integration with that core [3]." A precise definition is given in [5]. We aggregate CBSA's into larger metropolitan areas based on airport sharing. The Bureau of Transportation Statistics Master Coordinate table lists, for each airport code, the names of locations served by the airport [12]. We use name matching to identify the CBSAs served by a given airport. Since sometimes two CBSAs are served by the same airport, we define a city by using the following algorithm. We designate each CBSA as a *population unit* and then recursively aggregate any two population units served by the same airport into a single population unit, until no two population units are served by the same airport. The population units remaining at the end of the algorithm are the cities in our model. Thus, each city contains one or more CBSAs. From this point on, whenever we say city, we are referring to these CBSA aggregates.

Each CBSA contains one or more counties. The Census Bureau provides data describing the counties in each CBSA [3]. To get populations for each city, we use the Census Bureau's estimates of population for each CBSA for April 1st 2000 [7].

For workflow data from one city to another, we use the Census Bureau's County-To-County Worker Flow Files, which contain the results of a work travel questionnaire included in the 2000 Census [6, 4]. In particular, for every ordered pair of counties (o_A, o_B), the data contains the number of people who indicated that they live in county o_A and work in county o_B. We assume that each person who indicated that they live in o_A and work in o_B commutes daily from o_A to o_B. To compute the daily workflow from city C_A to city C_B, we sum over all county pairs (o_A, o_B) such that $o_A \in C_A$ and $o_B \in C_B$ of the workflow between o_A and o_B.

For airline travel data, we use the Bureau of Transportation Statistics Origin and Destination survey for all quarters of 2007, which contains a 10 % sample of all itineraries between U.S. cities [13]. The stops on an itinerary are listed using standard airport codes. As previously stated, we use the Bureau of Transportation Statistics Master Coordinate table to identify the city for a given airport code [12].

An itinerary often includes temporary stop-overs at intermediate airports. For example, a traveler going from Austin to New York may have an intermediate stop-over in Chicago. We count such a traveler as a single traveler from Austin to New York, not as one traveler from Austin to Chicago, and a second traveler from Chicago to New York. In other words, to count the number of travelers from city C_A and city C_B, we count the number of itineraries with origin in C_A and final destination in C_B. We sum over all quarters of 2007 to get the total number of travelers from city C_A to city C_B, and divide by 365 to get the number of daily airline travelers from C_A to C_B. We assume the number of airline travelers from C_A to C_B to be constant.

2.2 Disease Model Parameters

The parameters of the disease model described above are given in Table 1 of the main article. For the supplement, we used earlier estimates of H1N1p parameters [15]. For example, γ is set to $\frac{1}{6}$, corresponding to an estimated average infectious period of 6 days [1]. Similarly, ν and μ are set to $\frac{1}{3}$ and $\frac{1}{2}$, respectively. Given estimated values for R_0, we use the equation $R_0 = \frac{\beta}{\gamma}$ to determine corresponding values for β [10]. We assume that individuals that seek antivirals do so immediately upon leaving the asymptomatic compartment.

For all of the optimization runs presented in the article and supplement (unless indicated otherwise), the initial conditions are 100,000 infected individuals distributed across cities proportional to population sizes, 31 million AV courses already distributed pro rata among the cities and an additional federally-held stockpile of 50 million courses available for distribution.

3 Comparison of Model Output to Epidemiological Data

Figure 1 depicts the order in which cities are predicted to experience outbreaks in the model versus actual CDC case reports [8]. The figure is produced assuming an initial condition of 1000 cases in Mexico City and no antiviral treatment throughout the epidemic. For this analysis, we extended our city network model to include 29 of the largest Mexican cities in addition to the 100 largest U.S. metropolitan areas; connections between U.S. and Mexican cities were parameterized using airline data for the months of March and April [14], but Mexican cities were not connected to each other. U.S. states are listed on the vertical axis roughly in the order that the model predicts states should experience outbreaks; and the numbers listed along the horizontal axis represent numerical order in which states experience outbreaks. For each state, we used the date at which its 100th H1N1 case was reported by the CDC as a proxy for the date of the initial outbreak in the state [8]. The green bars indicate the order in which states experienced outbreaks (based on first 100 reported cases). For example, Illinois was the first state with 100 reported infections, and it has a green dot in the first column. Wisconsin, Arizona, and New York reported their 100th infection on the same day, so they have green bars spanning ranks 3 through 5. Since the disease model is stochastic, the order of infection in the model is random. The color on column j of each row indicates the probability that the state is the jth to be infected in the model and red dots represent the mean ordering for the state. For example, Nevada, on average, is the ninth state to be infected, as indicated by the red dot in Nevada's row. However, Nevada appeared as early as 6th and as late as 15th in simulations.

There is some qualitative agreement between the model and the data (Figure 1). However, a more robust comparison of the model to data would be desirable. Previous studies have demonstrated the importance of workflow and airtravel data to the spread of influenza [2, 16], including the early global spread of H1N1 [11]. The reported infection order is highly dependent on reporting and monitoring by the states. In our analysis, we attempt to reduce some of the noise by using the 100th case as the indicator of an outbreak. Infection order is also, in general, highly sensitive to small perturbations in the data; a single state can move several positions by altering a few reported cases.

4 Sensitivity Analysis

In this section, we analyze the sensitivity of the optimal policies reported in the main article to the value of R_0. Specifically, we calculate the optimal policies under low ($R_0 = 1.1$) and high estimates ($R_0 = 2.1$) recently reported [15, 9]. At the low value of R_0, the initially available 31 million AV

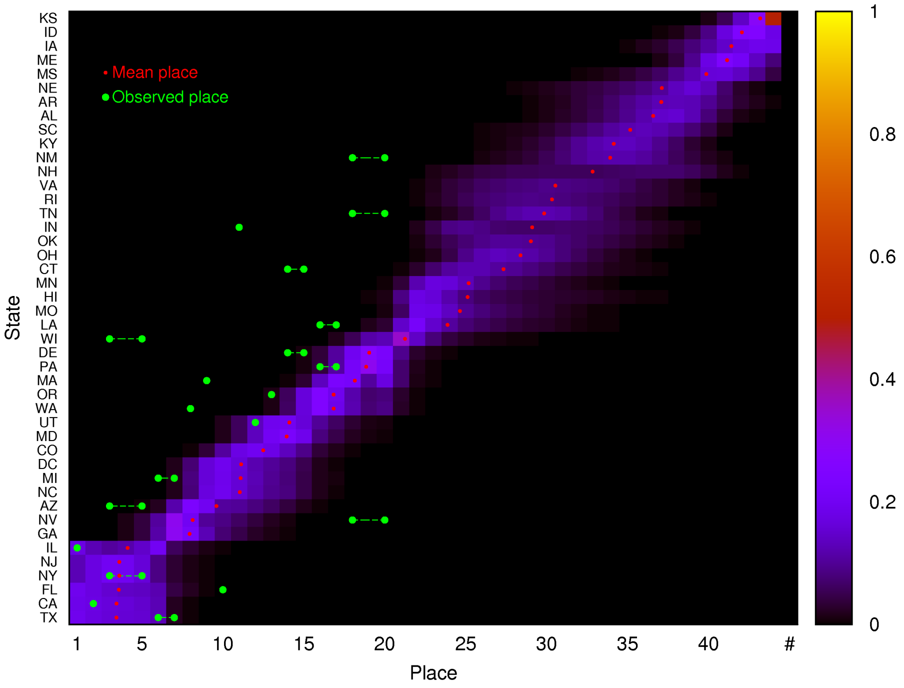

Figure 1: Comparison of model predictions to H1N1 data. The background color indicates the probability distribution of infection order as predicted by the model. The green bars indicate empirical infection order, as estimated by the timing of the 100th cases reported in the daily CDC update [8]. States are sorted by increasing mean infection order, as predicted by the model. The mean infection order for each state is depicted by a red dot in the corresponding row.

courses are sufficient to control the disease. That is why the optimized policy and all of the simple policies perform exactly the same in Figure 2a. At the high value of R_0, the optimizer outperforms all simple strategies. Moreover, the simple 5M release strategy is no longer the best of the simple strategies, and performs poorly across a wide range of uptake values. Larger quantities of antivirals are necessary to reign in the more explosive epidemics predicted to occur at $R_0 = 2.1$.

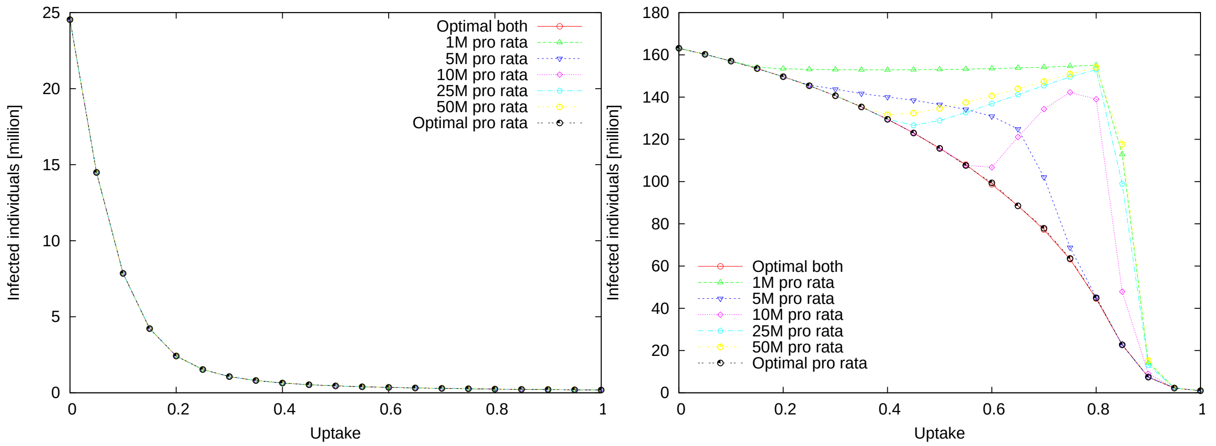

(a) (b)

Figure 2: Optimizer Performance. (2a) $R_0 = 1.1$. (2b) $R_0 = 2.1$. At a low value of R_0, the initially available 31 million antiviral courses are enough to control the disease, and all policies have the same performance. At a high value of R_0, the optimizer outperforms all simple policies.

Figure 3 depicts the optimized policies at the low and high values of R_0. At $R_0 = 1.1$, every policy is as good as the next, which is demonstrated by the seemingly random distribution schedules (Figure 3a) and random performances of various options during the first month (Figure 3b). At $R_0 = 2.1$, as the uptake increases from 0.50 to 0.95, the optimized policies delay and spread the release of antivirals so that they are available throughout the whole course of the epidemic. Even at high uptake values (0.45–0.85), the optimizer strongly discriminates between various release schedules (Figure 3d).

5 Supplemental Results

5.1 Simple Pro Rata Policies

Figure 4 shows the epidemic curves of five simple pro rata distribution policies in comparison to the optimized policy and a policy without intervention. The simple policies have a regular distribution of $1, 5, 10, 25$, or 50 million courses of antivirals every month, until the available stockpile of 50 million courses is depleted. The number of susceptible and recovered individuals at the end of each simulation (one year) shows that the optimized and the simple 5M policies perform better than all other policies. Under all suboptimal policies, antivirals are depleted mid-epidemic (the antiviral curve drops below the infected curve), and thus antivirals cannot mitigate the spread. The optimal policies release antiviral courses just in time, and thereby avoid wastage early on when there would not be sufficient numbers of cases to use available courses. In fact this delay allows the optimal policy under high levels of wastage (2 months half-life) to perform as well as the optimal policies under no wastage, as discussed in the main article.

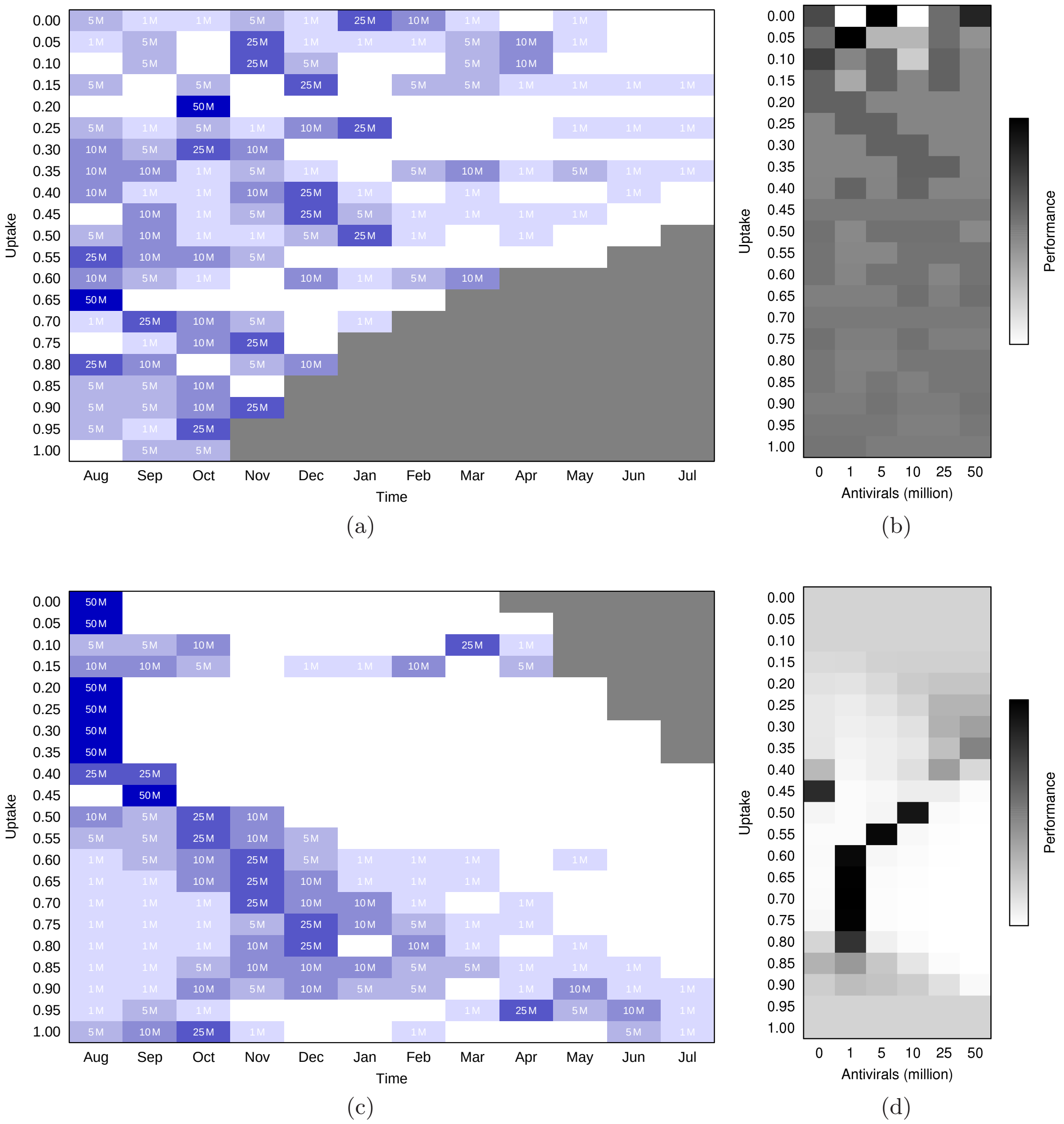

Figure 3: Optimized Policies. (3a) Optimized pro rata distributions for $R_0 = 1.1$. (3b) Performance of actions for the first (August) distribution for $R_0 = 1.1$. (3c) Optimized pro rata distributions for $R_0 = 2.1$. (3d) Performance of actions for the first (August) distribution for $R_0 = 2.1$. For the low value of R_0, almost any release strategy performs equally well. On the other hand, for the high value of R_0, the optimizer strongly discriminates between different policy options. For high R_0, we again see that as the uptake increases from 0.50 to 0.95, the optimizer delays and thins the bulk distribution of antivirals considerably.

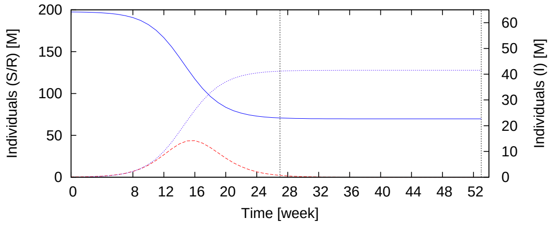

(a) No intervention.

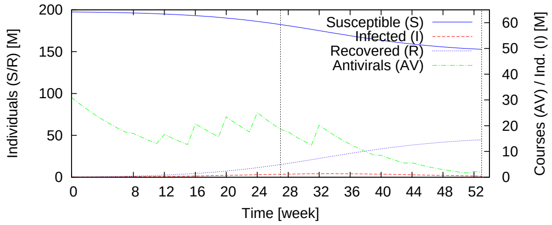

(b) Optimal policy.

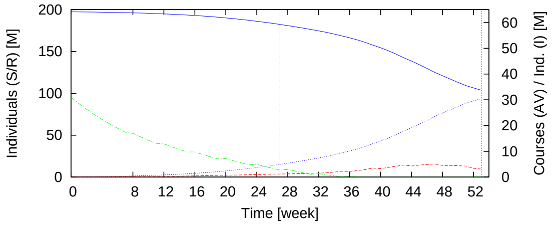

(c) Simple policy 1M.

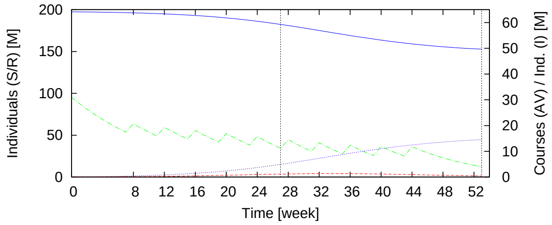

(d) Simple policy 5M.

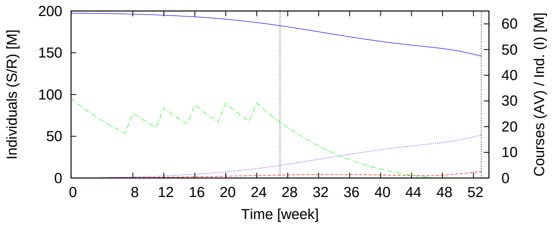

(e) Simple policy 10M.

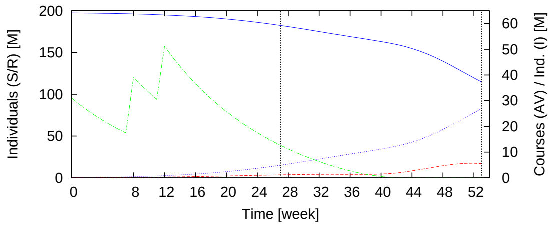

(f) Simple policy 25M.

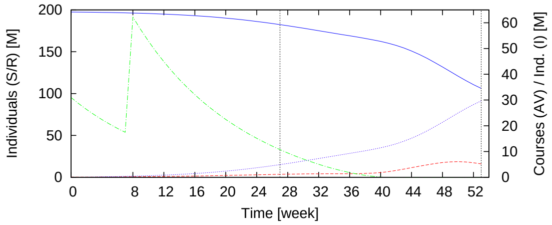

(g) Simple policy 50M.

Figure 4: Comparing simple pro rata distributions to no intervention and the optimized policy. The simple policies distribute the specified amount of antiviral courses each month proportional to population size. These curves were initialized with 100,000 infected individuals distributed among all cities proportional to population size, a total stockpile of 50 million antiviral courses available for distribution, and an additional 31 million courses already distributed pro rata to cities. The uptake value was $U = 0.5$, and the half-life (or misuse) for distributed antivirals was 2 months. The dashed vertical lines signify the half year and one year marks, respectively.

5.2 Prevalence-Based Distributions

For $R_0 = 1.6$ and all other parameters as given in Table 1 of the main article, Figure 5 compares the performance of simple prevalence-based distribution schedules with simple pro rata and optimized distribution policies. In all cases, the simple pro rata distribution beats the corresponding simple prevalence-based distribution. In addition, all simple prevalence-based distribution policies perform significantly worse than the optimized policies. This, along with the fact that few prevalence-based actions are included in the optimized mixed-mode policies (described in the main article), strongly suggests that pro rata distributions will be generally more effective than prevalence-based distributions.

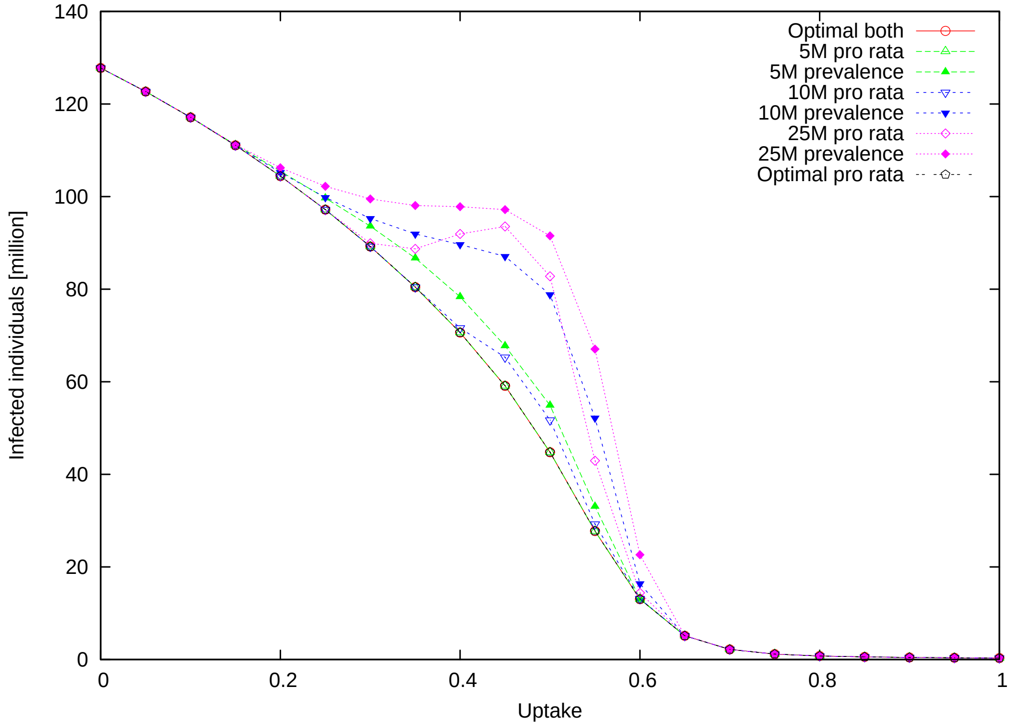

Figure 5: Prevalence-Based Policies. Simple prevalence-based distribution schedules release a fixed number of antivirals each month to cities, proportional to the current number of symptomatic infected individuals, until the stockpile is emptied. Each prevalence-based schedule is outperformed by the corresponding pro rata distribution policy and by all optimized policies.

5.3 Six Month Horizon

Figure 6 shows the optimal policies derived by the optimizer assuming a shorter time horizon. The optimizer rewarded policies based on the cumulative number of infected individuals at six months after the initial outbreak, in contrast to one year horizon described in the main article. For the shorter time horizon, at low rates of uptake, the optimal policies tend to release all antiviral courses at the outset; in contrast, the optimal year-long policies involve more delayed and extended distributions. Figure 4 illustrates that, when all 50M courses are released at the start, cities will run out of antivirals within 35 to 45 weeks of disease spread. Therefore, while an early release schedule can significantly lower final prevalence at six months, it cannot do so out to 12 months.

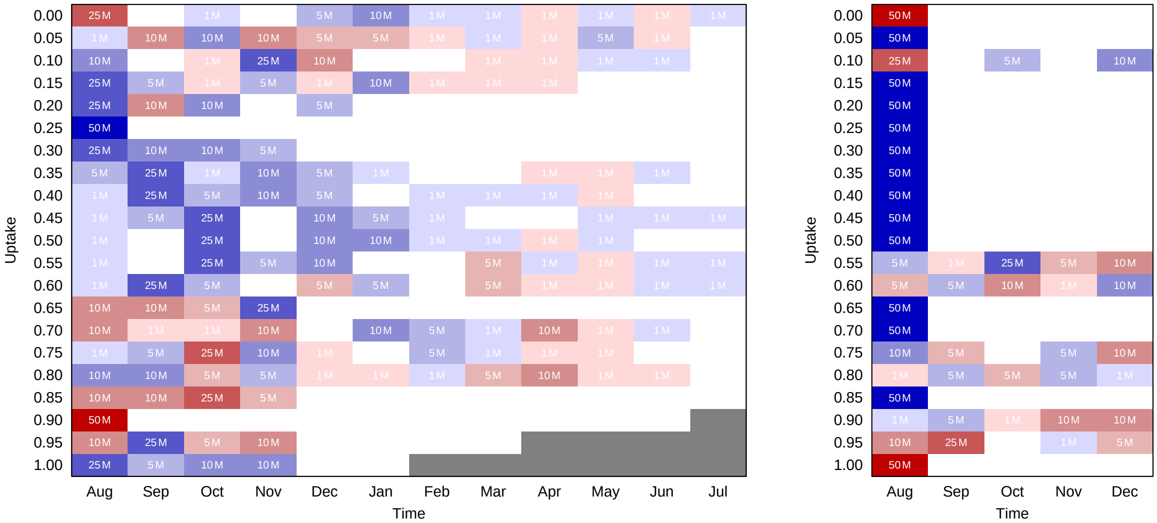

(a) Optimal policies for pro rata and prevalence-based distributions with 12 months and 6 months cutoff.

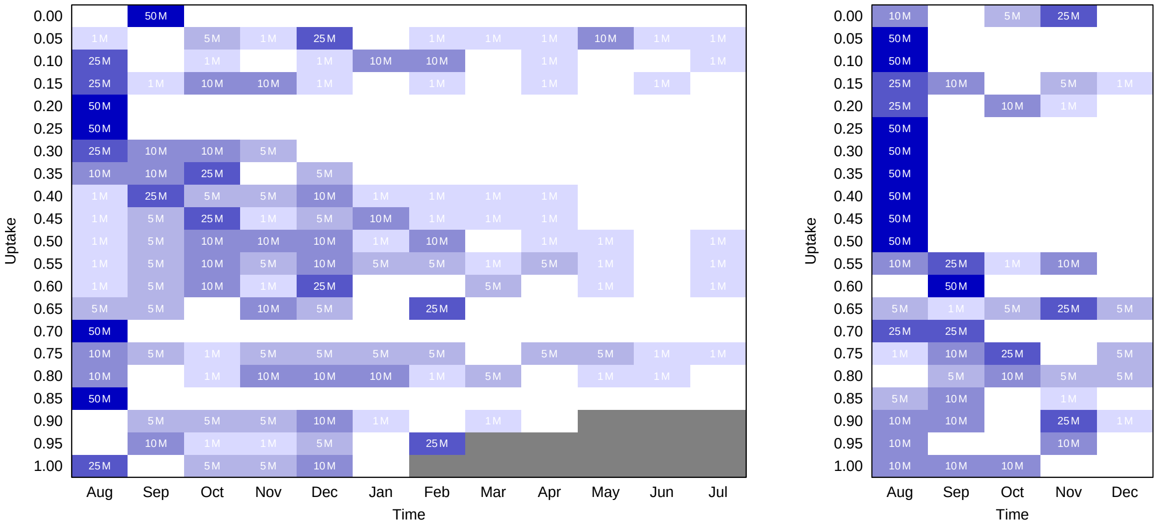

(b) Optimal policies for pro rata distribution with 12 months and 6 months cutoff.

Figure 6: Optimized policies at 12 and 6 month cutoffs. The optimizer choses policies based on total number of infected individuals (minus those effectively treated by antivirals) after 12 months or 6 months, respectively. The resulting optimal policies differ significantly in structure.

6 Complete Epidemic Horizon

We have also derived optimal policies assuming indefinite horizons, i. e., the optimizer allows simulation to continue until H1N1 disappears entirely and rewards policies based on total cases throughout the entire epidemic. This pertains to a scenario where effective vaccines do not become available and antivirals continue to be one of the primary intervention options.

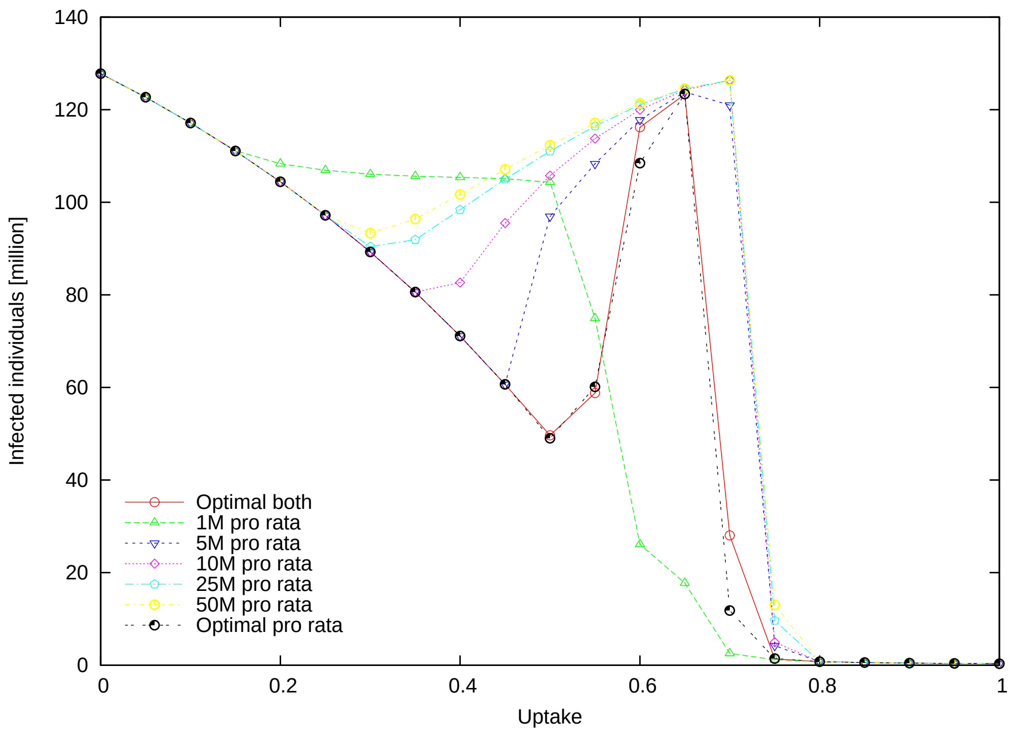

Figure 7: Optimizer performance for complete epidemics. The optimizer performs well at all uptake values, except those from 0.55 to 0.65. If antivirals are used, the effective R_0 at these uptake values hovers just above 1, leading to protracted epidemics. Effective control of these long epidemics requires extended duration small release schedules that go deep into the policy tree, which the UCT algorithm is unable to find given the specified tree structure.

The optimizer performs well at all uptake values, except those from 0.55 to 0.65, inclusive (Figure 7). Around these levels of uptake, antivirals reduce the effective R_0 of the disease to just above 1, leading to extraordinarily long epidemics. If the epidemic is long, the policy tree searched by the optimizer becomes very large. Indeed, the policy tree is not only large, but lopsided, since branches that release few antivirals lead to deep subtrees while branches that release large amounts of antivirals have short subtrees. Using our particular policy tree structure, the UCT algorithm is unable to find the deep, long policies required to perform well at these uptake values.

We compare the dynamics of the disease under the fixed 1M and fixed 5M release schedules, at a higher level of uptake ($U = 0.6$) than occurring today (Figure 8). The 5M policy contains the disease well for the first 18 months, however, at the 18 month mark, antivirals are no longer available. The disease has not been eradicated and most of the population remains susceptible. Thus, a full-fledged epidemic ensues at 24 months. On the other hand, the 1M policy releases antivirals much more slowly. In this case, the slow release of antivirals ensures antivirals are available throughout the entire course of the disease, containing the disease consistently throughout the epidemic period.

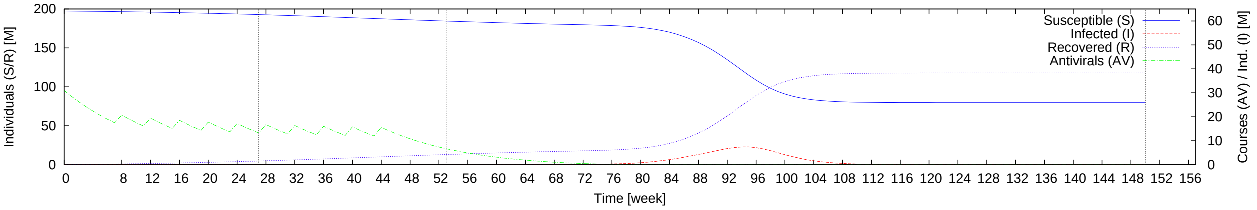

(a)

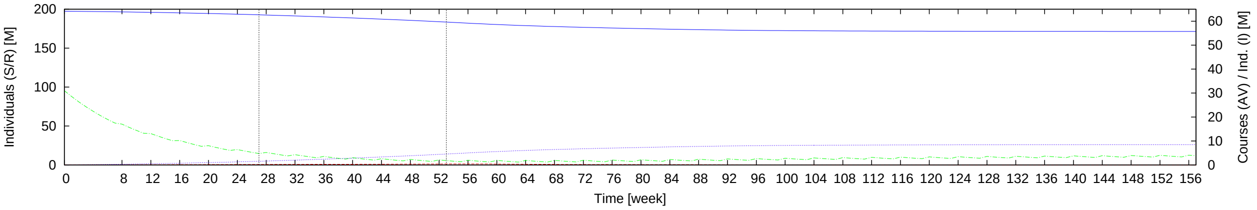

(b)

Figure 8: Disease dynamics under complete epidemic antiviral policies. (8a) Disease dynamics for a policy of releasing 5M antivirals for 10 months. (8b) Disease dynamics for a policy of releasing 1M antivirals for 50 months. The 5M policy contains the disease well for 18 months, but all antivirals are used and we see a full-fledged epidemic at 24 months. On the other hand, the slow release of the 1M policy ensures antivirals are available for long enough to mitigate the epidemic. These assume an uptake of 60 %.

Figure 9 depicts the optimized policies for the complete epidemic. At low uptake values, 0 to 0.25, few infected individuals seek treatment and so all distribution policy are equally poor, as indicated by the monocolor gray lines at the top of Figure 9c. As the uptake increases, 0.25 to 0.55, it becomes important to prevent antiviral wastage, and the optimized policies delay the release of antivirals and reduce the quantities released in each month. At uptakes between 0.60 and 0.70, the optimizer is unable to find the long policy going deep into the policy tree required to control the disease. At uptake values higher than 0.70, an overwhelming fraction of infected individuals seek treatment and the disease can be effectively eradicated by the optimized release schedules.

7 Visualization

Our epidemic visualizations (Video S1) depict the progression of epidemics in each of the 100 U.S. cities in our city network model. The areas of the circles representing cities are proportional to population sizes. Inside each circle, we use a smaller circle to represent the cumulative number of infections within each city. Around each circle, we use a blue ring to represent the number of antiviral courses available in each city. Both the inner infection circle and the outer antiviral ring have areas proportional to the respective number of individuals (or courses).

The color of the inner circle changes from red to black as the effective R (given by R_0 times the fraction of susceptibles) in each city drops below 1. At this point, disease spread in that city has reached its peak and the disease can be expected to die out shortly. The color of each city changes from yellow to gray as the city's local cache of antivirals is depleted, i. e., the fraction of antiviral courses over city population drops below 0.1 %. All the simulations are initialized using 100,000 cases distributed proportional to population size.

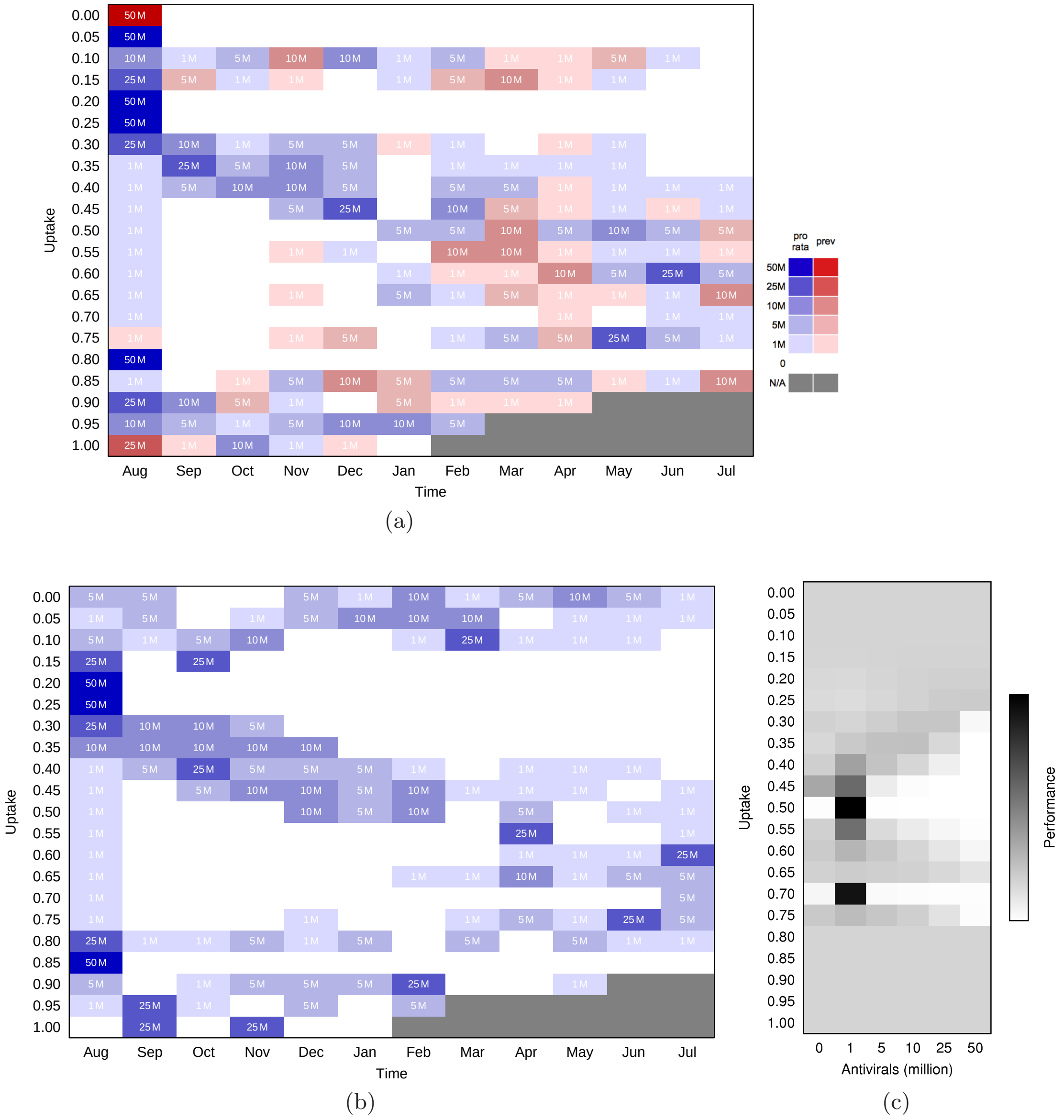

Figure 9: Complete Epidemic Optimized Policies. (9a) Optimized policies combining prevalence-based (red) and pro rata distributions (blue). Each row gives the optimal sequence of actions for a given value of uptake. (9b) Optimized pro rata distributions. (9c) Performance of actions for the first (August) distribution. Darkness indicates how many times an action was visited during the optimization routine. As the uptake increases from 0.30 to 0.55, the optimized policies increasingly delay and reduce the release of antivirals, so that antivirals are available throughout the epidemic.

7.1 No Intervention

Without intervention, the disease spreads rapidly and the epidemic runs its course by week 40.

7.2 Low Uptake

We consider two intervention policies at a plausible uptake value of 25 %. The first policy does not release any antivirals in addition to the initial 31M courses already held by states. This policy initially slows the disease somewhat. When the antiviral courses are depleted through treatment and wastage, the epidemic progresses as in the no intervention case.

The second policy releases an additional 5M courses per month for 10 months. Although antivirals are available to the cities throughout the entire period, the epidemic ultimately surges because of low antiviral uptake.

7.3 High Uptake

We consider three intervention policies at a higher uptake value of 50 %, which is unlikely without a major public health effort to increase treatment rates. Epidemic curves for some of these policies are included in the main article. The first policy does not release any antivirals in addition to the initial 31M courses released to the states. While antivirals are available, the disease progression is considerably slower than that observed for an uptake of 25 %. However, when the supply is depleted, there is an epidemic surge reaching proportions observed in the prior simulations.

The second policy distributes an additional 5M courses per month for 10 months, in addition to the previously distributed 31M courses. As for 25 % uptake, this policy guarantees antiviral availability throughout the whole course of the simulation. The disease progression is slowed considerably, and no city reaches an effective reproductive ratio of less than 1 at the end of twelve months. This indicates that the 5M policy effectively mitigates the epidemic throughout the first year.

The third policy distributes 25M courses per month for 2 months, in addition to the previously distributed 31M courses. While the large distributions maintain antiviral availability for several months, availability is quickly depleted due to wastage. Once antivirals are no longer available, the epidemic spreads rapidly.

References

[1] Interim guidance for clinicians on identifying and caring for patients with swine-origin influenza A (H1N1) virus infection.

[2] J. S. Brownstein, C. J. Wolfe, and K. D. Mandl. Empirical evidence for the effect of airline travel on inter-regional influenza spread in the united states. *PLoS Medicine*, 3:e401, 2006.

[3] Census Bureau. About metropolitan and micropolitan statistical areas. http://www.census.gov/population/www/metroareas/aboutmetro.html.

[4] Census Bureau. County-to-county worker flow files. http://www.census.gov/population/www/cen2000/commuting/index.html.

[5] Census Bureau. Current lists of metropolitan and micropolitan statistical areas and definitions. http://www.census.gov/population/www/metroareas/metrodef.html.

[6] Census Bureau. Journey to work and place of work 2000.
http://www.census.gov/population/www/socdemo/journey.html.

[7] Census Bureau. Population estimates for core based statistical areas.
http://www.census.gov/popest/metro/CBSA-est2007-annual.html.

[8] CDC. H1N1 flu daily updates.
http://www.cdc.gov/h1n1flu/updates/.

[9] C. Fraser, C.A. Donnelly, S. Cauchemez, W.P. Hanage, M.D. Van Kerkhove, T.D. Hollingsworth, J. Griffin, R.F. Baggaley, H.E. Jenkins, E.J. Lyons, T. Jombart, W.R. Hinsley, N.C. Grassly, F. Balloux, A.C. Ghani, N.M. Ferguson, A. Rambaut, O.G. Pybus, H. Lopez-Gatell, C.M. Alpuche-Aranda, I.B. Chapela, E.P. Zavala, D.M.E. Guevara, F. Checchi, E. Garcia, S. Hugonnet, C. Roth, and The WHO Rapid Pandemic Assessment Collaboration. *Science*, 5934:1557–1561, 2009.

[10] M. J. Keeling and P. Rohani. *Modeling Infectious Diseases*. Princeton University Press, 2008.

[11] K. Khan, J Arino, W. Hu, P. Raposo, J. Sears, F. Calderon, C. Heidebrecht, M. Macdonald, J. Liauw, A. Chan, and M. Gardam. Spread of a novel influenza A (H1N1) virus via global airline transportation. *New England Journal of Medicine*, 361:212–214, 2009.

[12] Bureau of Transportation Statistics. Airport master coordinate data.
http://www.transtats.bts.gov/Fields.asp?Table_ID=288.

[13] Bureau of Transportation Statistics. Origin and destination survey, all quarters of 2007.
http://www.transtats.bts.gov/Fields.asp?Table_ID=289.

[14] Bureau of Transportation Statistics. T-100 international market (all carriers), 2007.
http://www.transtats.bts.gov/Fields.asp?Table_ID=260.

[15] B. Pourbohloul, A. Ahued, B. Davoudi, R. Meza, L.A. Meyers, D.M. Skowronski, I. Villasenor, P. Kuri, F. Galvan, P. Cravioto, J. Trujillo, D.J. Earn, J. Dushoff, D. Fisman, J. Edmunds, N. Hupert, S.V. Scarpino, D.M. Patrick, and R.C. Brunham. *Influenza and Other Respiratory Viruses*, page in press, 2009.

[16] C. Viboud, O. N. Bjørnstad, D. L. Smith, L. Simonsen, M. A. Miller, and B. T. Grenfell. Synchrony, waves, and spatial hierarchies in the spread of influenza. *Science*, 312:447–451, 2006.
